# Fermentation of glycerol by a newly discovered anaerobic bacterium: adding value to biodiesel production

**DOI:** 10.1111/1751-7915.12709

**Published:** 2017-03-23

**Authors:** María Hidalgo, Elena Puerta‐Fernández

**Affiliations:** ^1^ Departamento de GenéticaUniversidad de SevillaAvda. Reina Mercedes, 641012SevillaSpain

In the last years, there is a rising interest in substituting fossil‐derived fuels by biofuels, due mostly to environmental reasons and the finite nature of the former ones. Biodiesel, together with bioethanol, are the two more volumetrically produced biofuels worldwide. Moreover, in the last decade, biodiesel production in Europe contributed to more than 80% of global biodiesel production (Demirbas and Balat, 2006), with an estimated production of over 10 million tons in 2015 and a production capacity of 23 million tons (Patil *et al*., 2017).

By definition, biodiesel is any liquid fuel derived from organic acids, such as vegetable oil or animal fat, that can be used in standard diesel engines. It can be used either alone or blended with petro‐diesel in different proportions. Biodiesel consists of long‐chain alkyl esters and is typically made by chemically reacting lipids with an alcohol. During its synthesis, a considerable amount of glycerol is produced. Glycerol (1, 2, 3‐propanetriol) is a simple trivalent alcohol that is naturally found as the backbone of animal and plants triglycerides. Although it has wide applications in different industries (food, pharmaceutical, cosmetics, tobacco…), its increased co‐production in biodiesel industries has made it a waste product instead of a valuable co‐product; moreover, the glycerol obtained during biofuel synthesis cannot be directly use in any industrial application due to the impurities that contains. On the other hand, its chemical composition makes it a better feedstock in yield terms than sugars for fermentation into reduced products, such as ethanol or H_2_ (Murarka *et al*., 2008).

In this issue of Microbial Biotechnology, Patil *et al*. (2017) describe the use of a newly discovered anaerobic bacterium that ferments glycerol. This anaerobic bacterium, *Anaerobium acetethylicum*, converts glycerol into two interesting biofuels: ethanol and hydrogen, with very little amounts of undesired co‐products. Bio‐ethanol is considered an alternative to fossil fuels, being renewable and with potential to reduce particulate emissions (Hansen *et al*., 2005). As mentioned before, it is the most common biofuel produced worldwide and can be used in gasoline engines, either in its pure form, or blended with gasoline. Hydrogen is a very interesting biofuel. In terms of mass, its energy content is higher than any other fuel, and its use in fuel cells ensures production of pollution‐free electricity. However, hydrogen is still mostly produced by steam reforming from hydrocarbons, although there is a huge research interest in its bio‐based production, using either photosynthetic organisms, such as *Rhodobacter capsulatus* or *Chlamydomonas reinhardtii* (Scoma *et al*., 2012; Abo‐Hashesh *et al*., 2013), or by anaerobic fermentation of sugars with different microbes, such as strains from the *Clostridium* or *Enterobacter* genera (Hung *et al*., 2011). Conversion of glycerol into these two valuable biofuels seems therefore an excellent way to add value to the well‐established biodiesel industry.

*Anaerobium acetethylicum* was recently isolated from sludge samples obtained from a biogas reactor at Germany. It was described as able to ferment gluconate, although the authors also reported growth on glycerol under strict anoxic conditions (Patil *et al*., 2015). It has been taxonomically classified into the order Clostridiales, and its genome has been sequenced (Patil *et al*., 2017). In their current article, the authors described optimal conditions for glycerol fermentation to ethanol and hydrogen, with very low production of other fermentation products (Patil *et al*., 2017). *A. acetethylicum* can grow in up to 1500 mM of glycerol, in the total absence of complex organic supplements, and the maximum ethanol production observed was 60 mM. As mentioned above, little production of undesirable co‐products (acetate, formate and propylene glycol) was observed, although the authors reported the presence of a fermentation product that they have not been able to identify so far. They have discarded several common fermentation products, such as butanol, propanol or butyrate, and the nature of the molecule remains still undiscovered.

Even though glycerol fermentation most often leads to 1,3‐propanediol (Homann *et al*., 1990), several bacteria have been described to ferment the surplus glycerol from biodiesel industries. Murarka *et al*. (2008) described the fermentation of glycerol to ethanol by an anaerobically grown *Escherichia coli* strain; they also reported H_2_ production, but they observed that H_2_ accumulation was detrimental for final ethanol yield. *Clostridium* has also been reported to ferment glycerol to butanol, although accumulation of co‐products was reported (Biebl, 2001; Dharmadi *et al*., 2006), and several environmental bacteria have been described to produce either ethanol or hydrogen from glycerol (Rossi *et al*., 2012; Marone *et al*., 2015). The main advantages that *Anaerobium acetethylicum* possess over these other bacteria are its higher glycerol tolerance and growth rate, lower co‐products formation and lower need of organic supplements in the media for optimal fermentation.

The authors report that *A. acetethylicum* degrades glycerol via glyceraldehyde‐3‐phosphate, which is further metabolized through glycolysis to ethanol and hydrogen. The enzymes implicated in the process were identified by proteome analysis, and the key enzymatic activities involved in the fermentation process were assayed in cell‐free extracts of glycerol‐grown cells. Moreover, activity of glycerol dehydrogenase, the first enzyme in the metabolic pathway proposed, was not detected in the cell extract of glucose‐grown cells, indicating specific expression during glycerol growth.

Even though *A. acetethylicum* production of ethanol and hydrogen is still far from being profitable in an industrial process, and consumption of glycerol should be improved, there are several items that could be easily implemented to optimize the process. The authors report a decrease in the medium pH during fermentation, dropping below the optimal pH described for this bacterium (Patil *et al*., 2015). A fermentation process with controlled pH should, *a priori*, improve growth and, therefore, glycerol consumption. Moreover, the authors describe an increase in biofuels production (both ethanol and H_2_) by increasing the headspace‐to‐culture volume ratio. This might be due to an inhibitory effect cause by H_2_ solubilization in the culture media. A feature similar to that has been described for anaerobic fermentation of glycerol by *E. coli* (Murarka *et al*., 2008), and it could be avoided by sparging the fermenter with nitrogen or argon during fermentation, or by early recovering of the produced H_2_. Ethanol production might also be improved by ethanol recovering, since the authors speculate of a possible inhibitory effect due to ethanol toxicity (Patil *et al*., 2017).

Production of waste residues is common in all industrial processes, and biofuel industries are not an exception. Biodiesel production results in surplus glycerol, whereas bioethanol production from plant biomass results in large amounts of lignin residues. These examples illustrate well the necessity of materializing the biorefineries concept, in a way similar to the classical petroleum refineries (de Jong and Jungmeier, 2015). The isolation of microbes able to convert waste into valuable products would ensure increasing process profits and means a step forward the achievement of the whole biorefinery concept.

## Conflict of interest

None declared.
